# Targeted therapies and adverse drug reactions in oncology: the role of clinical pharmacist in pharmacovigilance

**DOI:** 10.1007/s11096-018-0653-5

**Published:** 2018-05-21

**Authors:** G. Fornasier, M. Taborelli, S. Francescon, J. Polesel, M. Aliberti, P. De Paoli, P. Baldo

**Affiliations:** 1 Pharmacy Unit, CRO Aviano National Cancer Institute – IRCCS, Via F. Gallini 2, 33080 Aviano, PN Italy; 2 Unit of Cancer Epidemiology, CRO Aviano National Cancer Institute – IRCCS, Via F. Gallini 2, 33080 Aviano, Italy; 3 Scientific Directorate, CRO Aviano National Cancer Institute – IRCCS, Via F. Gallini 2, 33080 Aviano, PN Italy

**Keywords:** Adverse drug reaction, Italy, Oncology, Pharmacist, Pharmacovigilance, Safety, Targeted-therapies, Under-reporting

## Abstract

*Background* The majority of adverse drug reactions (ADRs) reported in the summary of product characteristics (SPCs) are based on pivotal clinical trials, performed under controlled conditions and with selected patients. *Objectives* (1) to observe ADRs in the real-world setting and to evaluate if the supervision of the pharmacist impacts on the management of ADRs and on the satisfaction of patients; (2) to sensitise health professionals and patients on the need to increase the reporting of ADRs, in compliance with Pharmacovigilance. *Setting* CRO Aviano, Italian National Cancer Institute. *Method* From February 2013 to April 2015, we conducted an observational study enrolling 154 patients (≥ 18 years) undergoing treatment with at least one of ten targeted-therapies included in the study. *Main outcome* ADR reporting in the real-world setting. Patient satisfaction with clinical pharmacist support. *Results* Reported ADRs in the real setting do not always correspond with data described in the respective SPCs. Unknown ADRs were also identified such as hyperglycaemia with lenalidomide and sorafenib; and hypomagnesaemia with bevacizumab. We also observed a 124.3% increase in spontaneous reports. *Conclusion* This study shows the high value of active pharmacovigilance programs, and our results might be a starting point for developing a randomised trial which should aim to demonstrate the impact of the pharmacist on improving patient’s adherence and in measuring the difference in ADRs reports in the different arms followed or not by the pharmacist.

**Electronic supplementary material:**

The online version of this article (10.1007/s11096-018-0653-5) contains supplementary material, which is available to authorized users.

## Impacts on Practice

- Pharmacovigilance studies are essential in oncology, because the under-reporting phenomenon is especially relevant in this field. In particular, physicians often underestimate the adverse reactions caused by oncological drugs, because they consider them common and they rather focus on efficacy of the drugs.
- Pro-active pharmacovigilance is important to improve spontaneous reporting that can generate new signals on adverse drug reactions (ADRs). These signals can lead the Competent Authority to make a decision on each single drug (alerts, recommends…).
- Real life conditions are needed to detect the real incidence of the ADRs;
- Individual patient monitoring improves their compliance, because patients receive more information and they are directly involved in treatment.

## Introduction

In recent years, anticancer treatments have seen an enormous evolution. Targeted therapies are a new generation of anticancer drugs which interfere with specific molecules that are involved in the growth, progression, and spread of cancer, and are expressed by specific cancer types. These drugs have introduced the concept of individually-tailored cancer treatment. The specific mechanism of action of target therapies should cause less toxic effects than conventional chemotherapy drugs [1]. The specific safety profile of each new drug, which is the information known about its safety at the time of marketing, has always been based on pivotal clinical trials. For this reason, the adverse drug reactions (ADRs) reported in the Summary of Product Characteristics (SPC) cannot be exhaustive [2–4], because the potential new drugs are tested under controlled conditions, with a small sample of selected patients, and often with a short follow-up time. The differences between selected patients enrolled in clinical trials and the real-world patients regard the characteristics of the latter [2]. Often, the real-world patients in oncology are represented by elderly patients which may be considered as a “special population”. Elderly patients are at a high risk of ADRs, medical errors, and drug interactions, because of comorbidities, polypharmacy, greater vulnerability, and age-related changes in pharmacokinetics and pharmacodynamics [4].

In everyday clinical practice, there is a significant list of side-effects that can be ascribed to targeted drugs; indeed, although they are different from the well-known side effects caused by chemotherapy, they can severely compromise patients’ quality of life, and can even cause discontinuation of therapy or changes in the therapeutic strategy [5].

Pharmacovigilance aims to improve patients’ safety through the detection of ADRs. Spontaneous reporting is the best method for highlighting adverse drug reactions outside clinical trials [6].

In oncology, under-reporting of adverse reactions is a common phenomenon, because ADRs are generally considered inevitable [7]. In addition, there is a relevant decrease of patient’s satisfaction during anticancer treatment as a consequence of ADRs. A shift in the trend of reporting ADRs by health professionals and patients is considered to be an important element to improve patient’s safety and adherence to targeted therapies. The involvement of patients in Pharmacovigilance reporting systems (PVRS) is a potential strategy to increase the knowledge of oncological ADRs, increase patient’s satisfaction, and reduce the phenomenon of under-reporting [8–10].

### Aims of the study

The main objective of this study was to observe the ADRs caused by ten anticancer targeted-therapies in the real-world oncology setting and to detect the differences with the ADRs reported in SPCs. As secondary outcome, this study investigated the role and the impact of the clinical pharmacist in reducing under-reporting of ADRs and in improving their management. Moreover, patients’ satisfaction with the support provided by the clinical pharmacist was investigated at the conclusion of the study.

### Ethics approval

The study was approved by CRO National Cancer Institute Ethical Committee (Project No. 199/Sc).

## Method

### Study design

The present study was conducted at the National Cancer Institute “Centro di Riferimento Oncologico—CRO” in Aviano, north-eastern Italy, between February 2013 and April 2015, enrolling 154 patients, aged 18 years and older. We performed a prospective, observational pilot study to evaluate the safety profile of 10 anticancer targeted-therapies. All patients were treated with at least one targeted-therapy at the doses reported in SPC. The study focused on eight oral drugs: erlotinib, everolimus, gefitinib, imatinib, lapatinib, lenalidomide, sorafenib, sunitinib, and two injectable drugs: bevacizumab and cetuximab. To avoid biases caused by concomitant administered drugs, we enrolled patients in monotherapy with the studied drugs (excluding cetuximab). Indeed, to remove the effect of earlier treatment, patients previously treated with other drugs underwent a wash-out period of 12 weeks. The recruitment phase started in February 2013 and lasted 18 months.

Eligible patients who fulfilled the inclusion criteria (age ≥ 18 years, patients treated at CRO of Aviano with at least one of the study drugs) and signed the informed consent, were interviewed by a clinical pharmacist using a structured questionnaire to collect data on socio-demographic characteristics, drug schedule, and ADRs. Particular attention was paid to the evaluation of patient’s interactions with the adverse events occurring during treatment, assessing whether patients had ever heard of PVRS, if they knew how it works, and if they had ever reported any ADRs to PVRS. Patients not using these ten target-therapies or not treated at CRO Aviano were excluded. Every 30 days, patients were contacted by a clinical pharmacist for the follow-up interview to evaluate their health condition and ADRs observed during therapy. Metabolism and nutrition disorders (hyperglycaemia, hypomagnesaemia, according to MedDRA dictionary) were also reported, since patients in treatment with target therapies have to perform routinely analysis every month. These interviews were conducted face-to-face or by telephone, according to the patients’ preferences, using a structured questionnaire (see additional downloadable material). In the final interview, patient’s compliance and satisfaction were further assessed. Patients’ compliance was also observed for oral drugs through the hospital pharmacy service. Indeed, we gave another medicine pack only if the patient had actually taken the drugs given before. Furthermore, in the final interview, patients were asked whether they appreciated the monthly monitoring, if they would have liked to be monitored after the study, and whether pharmacist monitoring impacted on the therapy adherence and management of ADRs. The answers to the final interview were anonymously collected by the nurses, in order to avoid biases. The ADRs observed were compared with those reported in SPC. We could not analyze the data concerning erlotinib, gefitinib, and lapatinib because there were < 10 enrolled patients.

ADR terminology was firstly classified according to MedDRA dictionary [11]. Information on ADRs were then analysed using the World Health Organization-Uppsala Monitoring Center (WHO-UMC) criteria and the Naranjo algorithm to evaluate whether they were connected to the drug or not (causality assessment) [12].

## Results

### Study population

Overall, 154 patients were enrolled (Table 1). The majority were female (n = 83, 54%) and were 65 years or older (n = 60, 39%) (median age: 61 years; interquartile range 52–69 years). The therapy with bevacizumab was the most frequent (n = 49, 32%), followed by Sunitinib (n = 27, 18%), and Sorafenib (n = 20, 13%). Therefore, the most common pathology was breast cancer (n = 34, 22%), followed by colorectal cancer (n = 31, 20%), and renal cell carcinoma (n = 25, 16%). Six patients were sequentially treated with two drugs (Sunitinib/Everolimus or Sunitinib/Sorafenib) and three patients with three drugs (Sunitinib, Everolimus, and Sorafenib). The majority of patients presented comorbidities (n = 110, 71%), and received oncology pre-treatment (n = 94, 61%). For the specific purpose to the pilot study, we collected and reported data relative to detection of both “known” ADRs (reported by SPCs [13–19]) and “unknown” ADRs (not listed in SPCs by market holders). We researched the “unknown” ADRs in Eudravigilance and Agenzia Italiana del Farmaco (AIFA) database in order to understand if there were other cases of these ADRs [20, 21].

**Table 1:** Distribution of 154 eligible and evaluable patients on Target-Vig, according to targeted therapy and selected characteristics

	N	%
*Gender*		
Male	71	46.1
Female	83	53.9
*Age (years)*		
< 55	51	33.1
55–64	43	27.9
≥ 65	60	39.0
*Pathology*		
Breast cancer	34	22.1
Colorectal cancer	31	20.1
Renal cell carcinoma	25	16.2
Malignant gastrointestinal stromal tumour	17	11.0
Multiple myeloma	13	8.4
Lung cancer	11	7.1
Liver cancer	7	4.5
Ovarian cancer	6	3.9
Thyroid cancer	6	3.9
Chronic myeloid leukaemia	2	1.3
Oral cavity cancer	1	0.6
Pancreatic neuroendocrine cancer	1	0.6
*Targeted*-*therapy*^a^		
Bevacizumab	49	31.8
Cetuximab	12	7.8
Erlotinib	8	5.2
Everolimus	15	9.7
Gefitinib	2	1.3
Imatinib	12	7.8
Lapatinib	7	4.5
Lenalidomide	14	9.1
Sorafenib	20	13.0
Sunitinib	27	17.5
*No. of targeted*-*therapies*		
1	145	94.2
2	6	3.9
3	3	1.9
*Pre*-*treatment*		
No	60	39.0
Yes	94	61.0
*Comorbidity*		
No	44	28.6
Yes	110	71.4

^a^As nine patients were treated with more than one therapy, the sum can exceed the total

### Detection of “known” ADRs

Compared to the values of ADRs reported in SPC, we observed (Table 2) a higher frequency of increased lacrimation (41.7%, 95% CI 13.8–69.6% vs. 1 to < 10% in SPC), and eyelid oedema (66.7%, 95% CI 40.0–93.3% vs. 1 to < 10% in SPC) among patients treated with imatinib. Patients being treated with sorafenib reported a higher frequency of neuropathy (40.0%, 95% CI 18.5–61.5%) and skin desquamation (35%, 95% CI 14.1–55.9%) than that reported in SPCs (i.e., 1 to < 10%). Many differences emerged with sunitinib: 96.3% of patients reported hypothyroidism (95% CI 89.2–100 vs. 1 to < 10% in SPC); 55.6% reported musculoskeletal pain (95% CI 36.8–74.3% vs. 1 to < 10%), 44.4% cited increased blood creatinine and abdominal discomfort (95% CI 25.7–63.2% vs. 1 to < 10%); 37.0% indicated hypertriglyceridemia and hypercholesterolaemia (95% CI 18.8–55.3% vs. 1 to < 10% and 95% CI 18.8–55.3% vs. 0.1 to < 1%, respectively). In addition, a higher frequency of myalgia/arthralgia and headache (42.9%, 95% CI 29.0–56.7% and 28.6, 95% CI 15.9–41.2, respectively vs. 1 to < 10% in SPC) was detected among patients treated with bevacizumab. Finally, a difference in the incidence of mucositis (58.3%, 95% CI 30.4–86.2% vs. 1 to < 10% in SPC) was observed among patients treated with cetuximab. We did not notice differences between the ADRs observed with everolimus compared to ADRs reported in the SPC.

**Table 2:** Detected differences between the percentage of treated patients expected to experience adverse drug reactions (ADRs) as reported in the Summary of Product Characteristics (SPC) and the observed cumulative incidence of ADRs among 154 patients on Target-Vig

	Follow up (months)	Expected %	Observed	
	Median (IQR)		N	% (95% CI)
*Bevacizumab (n *= *49)*	6.7 (3.9–7.8)			
Myalgia		[1–10]	21	42.9 (29.0–56.7)
Arthralgia		[1–10]	21	42.9 (29.0–56.7)
Headache		[1–10]	14	28.6 (15.9–41.2)
*Cetuximab (n *= *12)*	3.4 (2.6–5.2)			
Nausea		[1–10]	5	41.7 (13.8–69.6)
Asthenia		[1–10]	5	41.7 (13.8–69.6)
Mucositis		[1–10]	7	58.3 (30.4–86.2)
*Imatinib (n *= *12)*	16.2 (14.6–18.4)			
Eyelids oedema		[1–10]	8	66.7 (40.0–93.3)
Lacrimation increased		[1–10]	5	41.7 (13.8–69.6)
Joint swelling		[1–10]	6	50.0 (21.7–78.3)
*Sorafenib (n *= *20)*	4.9 (2.9–11.5)			
Neuropathy		[1–10]	8	40.0 (18.5–61.5)
Skin desquamation		[1–10]	7	35.0 (14.1–55.9)
Myalgia		[1–10]	9	45.0 (23.2–66.8)
Arthralgia		[1–10]	9	45.0 (23.2–66.8)
*Sunitinib (n *= *27)*	9.9 (3.9–14.2)			
Hypothyroidism		[1–10]	26	96.3 (89.2–100)
Musculoskeletal pain		[1–10]	15	55.6 (36.8–74.3)
Blood creatinine increase		[1–10]	12	44.4 (25.7–63.2)
Abdominal discomfort		[1–10]	12	44.4 (25.7–63.2)
Hypertriglyceridemia		[1–10]	10	37.0 (18.8–55.3)
Flatulence		[1–10]	8	29.6 (12.4–46.9)
Oral pain		[1–10]	8	29.6 (12.4–46.9)
Hypercholesterolaemia		[0.1–1]	10	37.0 (18.8–55.3)
Hyperglycaemia		[0.1–1]	7	25.9 (9.4–42.5)
Dental abscess		[0.1–1]	5	18.5 (3.9–33.2)

*CI* Confidence Interval, *IQR* Interquartile Range

### Detection of “unknown” ADRs

Table 3 summarises several “unknown” ADRs (which are not reported by respective SPCs released by market holders) observed during the study. Among the patients treated with lenalidomide, the unknown ADRs were: hyperglycaemia (50.0%) and hypercholesterolaemia (28.6%). The same ADRs (25.0 and 15.0%, respectively), together with hypertriglyceridemia (10.0%), were also observed with sorafenib. Finally, patients treated with bevacizumab developed hypomagnesaemia (12.2%), while patients treated with cetuximab developed neutropenia (25.0%).

**Table 3:** Detection of new (unknown ADRs) among 154 patients on Target-Vig

	Follow up (months)	Observed	
	Median (IQR)	N	% (95% CI)
*Bevacizumab*	6.7 (3.9–7.8)		
Hypomagnesaemia		6	12.2 (3.1–21.4)
*Cetuximab*	3.4 (2.6–5.2)		
Neutropenia		3	25.0 (0.5–49.5)
*Lenalidomide*	6.7 (4.2–9.3)		
Hyperglycaemia		7	50.0 (23.8–76.2)
Hypercholesterolaemia		4	28.6 (4.9–52.2)
*Sorafenib*	4.9 (2.9–11.5)		
Hyperglycaemia		5	25.0 (6.0–44.0)
Hypercholesterolaemia		3	15.0 (0.0–30.6)
Hypertriglyceridaemia		2	10.0 (0.0–23.1)

*CI* Confidence Interval, *IQR* Interquartile Range

We also observed several ADRs which are reported as “uncommon” in SPCs (data not shown). The uncommon ADRs observed were deep vein thrombosis with everolimus (n = 3, 20.0%), eye disorders like blepharitis and keratitis with cetuximab (n = 3, 25.0%), and a transient ischaemic attack (TIA) with sorafenib (n = 1, 5.0%). A Doppler echocardiography was performed and showed a right carotid artery stenosis of 30% and left carotid artery stenosis of 50%. It must be highlighted that, due to the small number of known cases of adverse effects, the dosage of the drugs was not revised. Thus, this could result in higher health risks for patients.

### Impact of this study on patient satisfaction and on under-reporting of ADRs

The results of satisfaction questionnaire (see downloadable additional material) showed that this approach was appreciated by patients. Indeed, almost all of the patients (99%) appreciated the presence of the clinical pharmacist in the cancer care team and 99% considered the clinical pharmacist’s role essential for improving the quality of pharmaceutical care and patient’s adherence to the therapy. Furthermore, 96% of patients immediately contacted the pharmacist if there were any changes in the treatment or ADRs; moreover, 98% of patients would have wanted to be monitored after the end of the study. The adherence was not measured; however, through the clinical pharmacy service (direct hospital dispensation of drugs to outpatients) we observed that enrolled patients (95%) effectively took the entire medicine pack before taking another one. Comparison between pharmacovigilance reports received at CRO Aviano, National Cancer Institute, in 2013 (start of the study) and 2015 (end of the study) showed an increase (124%) of spontaneous reports (37 vs. 83 individual case reports).

## Discussion

The real conditions of clinical daily practice are needed to detect all of the possible “real” toxicities imputable to drugs, because ADRs reported in SPCs are essentially based on clinical trials. Patients often present comorbidities, polypharmacy, a greater vulnerability, and age-related changes in pharmacokinetics and pharmacodynamics. Indeed, the self-administration of oral drugs increases the risk of ADRs because of the improper use of drugs [22]; consequently, patients often suspend or self-regulate the dose of the drug when they experienced an important adverse drug reaction, with a possible decrease of therapy’s effectiveness. Furthermore, ADRs are the major problem of pharmacovigilance, especially in the oncology setting, as the anticancer drug toxicity is considered common, and a normal consequence of these therapies [23]. Health professionals do not report ADRs because they generally think that there could be negative consequences for the patients’ therapy if they fill out PV reports or complaints by the pharmaceutical industry [7, 24]. Moreover, they often have several doubts about how to appropriately complete the PV report. Often, patients are not informed about the existence of the PV report and about the possibility of directly completing this report. Motivating health professionals and patients to report ADRs to improve the knowledge of pharmacovigilance activities is an effective approach against under-reporting [4].

In this study, we showed the importance of pro-active pharmacovigilance in order to efficiently highlight adverse drug reactions, and the fundamental role of the pharmacist in ensuring patient’s safety. Most patients (71%) enrolled in the study presented comorbidities, and were therefore more vulnerable. The median age of patients enrolled in our study was 61 years old and the majority (54%) were female; in clinical trials, patients enrolled are young, male, and with no comorbidities.

Furthermore, it is also worth noting that even with a small number of patients, it has been possible to observe a difference in the incidence of some ADRs and to identify unknown ADRs. 28.6% of patients in monotherapy with bevacizumab had headaches but none had high blood pressure. Indeed, 42.9% of patients being treated with bevacizumab reported myalgia/arthralgia without disease progression. In patients treated with sunitinib, the collected data showed a significant difference in the incidence between ADRs observed in patients and those reported in SPC. All patients taking sunitinib followed the drug’s therapeutic scheme: 4 consecutive weeks, followed by a 2 week rest period. Thus, we considered the rest period to be a de-challenge and the drug re-intake as a re-challenge. We observed ADRs only during the drug intake period. Applying the PV principles, the correlation between ADRs reported and sunitinib was certain [12]. In particular, 96% of patients showed hypothyroidism during the treatment with sunitinib and 56% patients showed musculoskeletal pain without progression of the disease. We also observed 44% of patients with hypercholesterolaemia and hypertriglyceridemia, which improved during the interruption of sunitinib’s administration. In total, 8 of the 20 patients who took sorafenib showed neuropathy, so the drug had to be suspended because of this toxicity. Imatinib caused eyes toxicity; in particular, 67% of patients presented eyelid oedema, and 42% showed increased lacrimation. We observed unknown ADRs and searched for them in the Eudravigilance and AIFA databases to verify whether there were other reported cases other than ADRs [20, 21]. The results from our study showed that 12% of patients being treated with bevacizumab developed hypomagnesaemia and this adverse reaction was also reported in the Eudravigilance database. In the literature, this adverse effect is linked to anti-epidermal growth factor receptor (EGFR) monoclonal antibodies or to chemotherapy, but it is not linked to bevacizumab [25, 26]. Patients that used lenalidomide presented hyperglycaemia and hypercholesterolaemia as unknown adverse reactions. Those adverse effects have also been reported in the Eudravigilance database, but they could be associated with the concomitant use of dexamethasone. The unknown ADRs observed with sorafenib are also reported in the Eudravigilance database and we observed an improvement in adverse reactions with the suspension of sorafenib. Finally, although we observed neutropenia with cetuximab and there are some cases in the Eudravigilance and AIFA databases, the concomitant use of chemotherapy could affect this adverse effect. We also observed uncommon adverse effects with these ten targeted-therapies. These adverse events are not generally associated with the drugs and are consequently not reported to the regulatory agencies, causing a non-revised therapeutic administration of drugs. This can lead to serious health risks for the patient. It is important to report unknown and uncommon ADRs to the regulatory agency, in order to improve safety information about these new generations of drugs [2].

The results of the present study are also important for clinical practice. The monthly semi-structured interview allowed to understand if the drug had been taken correctly by the patient and intervened if there was any case of non-adherence. The review of Lam and Fresco [27] highlights that patient interviews and pill count are indirect measures used in routine clinical practice to verify patient’s non-adherence. Indeed, the monitoring of patients and the information gave them about the management of ADRs increase the satisfaction of patients and the dialogue between patients, health professionals, and pharmacists. Patients contacted the pharmacist if ADRs were present; if patients contacted their pharmacist before taking other substances (supplementations, herb, medical devices, etc.) or to understand how to take the drugs, the risk of interactions and adverse effects could decrease. Finally, the involvement of pharmacists in this study increased the pharmacovigilance reports from the beginning to the end of the study (37 vs. 83 individual case reports) [28].

### Strengths and limitations

The most important strength of the present study is that we observed the occurrence of ADRs in real-world conditions. Moreover, the structured interview, done every 30 days, allowed the close monitoring of ADRs. Study limitations are inherent to the observational nature of the study, which only permitted to retrieve a small number of adverse drug reactions. Furthermore, in regard to “known” ADRs, we used the data reported in specific SPCs as term of comparison of ADRs observed in the study, but obviously this can lead to potential biases correlated with the differences between the subjects recruited in the pre-registration trials and the population group participating to this study. So this may render to an impoverishment of the statistical design, and somehow arbitrary comparisons. Another limitation is the use of non-validated questionnaire forms. Regarding this, it is necessary to make a distinction between the methods used in the standard or “passive” pharmacovigilance activities—passive because based on the mere respect of reporting methods established by national or international legislation (e.g. CIOMS forms)—and methods used by “proactive” Pharmacovigilance studies—carried out with the educational purpose of citizens, patients, and health professionals in order to reduce underreporting of ADRs and detect potentially serious hazards for patients. To our knowledge, one of the future challenges involving PVRS will be the validation of questionnaires forms to be proposed as standard documentation during medication reviews activities and drug reconciliation.

Another limitation of the study was that blood and lymphatic system disorders (according to MedDRA dictionary) were underestimated because patients did not understand haematological exams, so we decided not to consider haematological toxicity in our analysis.

## Conclusion

During the study, we observed a difference of ADRs between the SPC and patients enrolled in the study as well as some unknown ADRs. This observational study also highlighted that there was an increase in pharmacovigilance reports, and therefore a decrease in the under-reporting phenomenon. This improved the information about ADRs of these ten targeted-therapies. Patient’s monitoring improved their satisfaction and decreased the autonomous suspension of drugs or self-regulation, and consequently adverse effects. Indeed, patients immediately contacted the pharmacist if there were changes in the treatment or ADRs. These results have given a high value to pharmacovigilance studies and the presence of clinical pharmacists in the treatment team to reduce the phenomenon of under-reporting. Results from the present study might be a starting point for developing a randomised trial which aims to confirm the impact of the pharmacist on improving patient’s adherence and in measuring the difference in ADRs reports with the intervention of the pharmacist.

## Electronic supplementary material

Below is the link to the electronic supplementary material.

Supplementary material 1 (DOCX 13 kb)

Supplementary material 2 (DOCX 13 kb)

Supplementary material 3 (DOCX 12 kb)
