# Supplementary material for: Targeted therapies and adverse drug reactions in oncology: the role of clinical pharmacist in pharmacovigilance
Source: Int J Clin Pharm. 2018 May 21;40(4):795–802. doi: 10.1007/s11096-018-0653-5 (PMC6132980; doi:10.1007/s11096-018-0653-5)
Supplement: Supplementary file 1 — Supplementary material 1 (DOCX 13 kb) [file 11096_2018_653_MOESM1_ESM.docx]

**First Interview**

1. Which of these drugs are you taking? erlotinib (Tarceva®), everolimus (Afinitor®), gefitinib (Iressa®), imatinib (Glivec®), lapatinib (Tyverb®), lenalidomide (Revlimid®), sorafenib (Nexavar®), sunitinib (Sutent®), and two injectable drugs: Bevacizumab (Avastin ®), cetuximab(Erbitux®)
2. DATE you started taking this drug: _________________________
3. Are you using other oncological drugs (outside of this list)? YES NO

If YES: Which drugs are you taking?

If YES: patient are not enrolled (excluded for cetuximab)

1. Did you use other oncological drugs before? YES NO

If YES:

a) Which drugs did you take?

b) Do you remember when you stopped them? DATE

1. Are you using other non-oncological drugs? YES NO

If YES: Which drugs are you taking?

1. Have you observed some Adverse drug reactions? YES NO

If YES: May you describe your ADRs?

1. Did you have these ADRs before you started (name of targeted-therapy)? YES NO
2. Did you speak about these ADRs with your physician or oncologist? YES NO
3. Do you know the ADRs reported in SPC? YES NO
4. Did your physician inform you that these drugs can cause some ADRs? Which ADRs did your doctor list? YES NO
5. Do you know what Pharmacovigilance is? YES NO
6. Do you know that you can report your ADRs to the Pharmacovigilance System? YES NO
7. Do you think that improving your knowledge about the ADRs caused by your drugs is useful? YES NO Why?
