# Supplementary material for: Targeted therapies and adverse drug reactions in oncology: the role of clinical pharmacist in pharmacovigilance
Source: Int J Clin Pharm. 2018 May 21;40(4):795–802. doi: 10.1007/s11096-018-0653-5 (PMC6132980; doi:10.1007/s11096-018-0653-5)
Supplement: Supplementary file 2 — Supplementary material 2 (DOCX 13 kb) [file 11096_2018_653_MOESM2_ESM.docx]

**Monthly interview**

1. Are you still taking (name of targeted-therapy indicated in first interview)? YES NO
2. If the answer is NO:

a) When did you stop taking it?

b) Reasons for stopping the drugs:

1. If the answer is YES:

a) Did you have difficulties in taking the drugs?

If YES: What difficulties?

b) Did you reduce the dosing by yourself? YES NO

If YES: Why?

c) Did you doctor reduce the dosing? YES NO

If YES: Why?

d) Do you ever forget to take your drugs?

e) Did you suspend your drugs? YES NO

If YES: When? Why? Did you inform your physician or oncologist? YES NO

f) Have you observed ADRs? YES NO

g) May you describe your ADRs?

h) Did you mention them in the first interview? YES NO

i) Have you observed an improvement of ADRs? YES NO

l) Did you use something to improve them? YES NO

If YES: What?

m) Did you speak about these ADRs with your physician or oncologist? YES NO

1. Did you report ADRs to the pharmacovigilance system? YES NO

If NO: Why?

1. Are you still using the same non-oncological drugs? YES NO

If NO: Why?

1. Are you using other new non-oncological drugs? YES NO
