# Supplementary material for: Targeted therapies and adverse drug reactions in oncology: the role of clinical pharmacist in pharmacovigilance
Source: Int J Clin Pharm. 2018 May 21;40(4):795–802. doi: 10.1007/s11096-018-0653-5 (PMC6132980; doi:10.1007/s11096-018-0653-5)
Supplement: Supplementary file 3 — Supplementary material 3 (DOCX 12 kb) [file 11096_2018_653_MOESM3_ESM.docx]

**Final interview**

1. Did you appreciate the monthly interview? YES NO
2. Did you contact the pharmacist before suspending the drugs? YES NO SOMETIMES
3. Did the monitoring make you feel directly involved in the treatment? YES NO
4. Would you like to be monitored monthly even at the end of the study? YES NO
5. Was the monitoring by the pharmacist useful to improve your quality of care and your adherence to the treatment? YES NO
6. Do you think that reporting ADRs is important? Why YES/NO?
